# Polysubstance-Induced Hepatotoxicity and the Role of Supportive Management

**DOI:** 10.7759/cureus.60649

**Published:** 2024-05-20

**Authors:** Nadim A Qadir, Luke Stachler, Anvit D Reddy, Gerardo Diaz-Garcia, Elisa Sottile

**Affiliations:** 1 Internal Medicine, University of Florida College of Medicine - Jacksonville, Jacksonville, USA

**Keywords:** drug-induced liver injury, nac-n acetyl cysteine, altered mental status, polysubstance use, supportive care and symptom management, cocaine

## Abstract

With the continued rise of polysubstance use throughout the country, it has been shown to affect a multitude of organ systems. Drug-induced liver injury (DILI) has been widely documented in its association with salicylates or acetaminophen and the utility of using N-acetylcysteine (NAC) for its hepatoprotective effects. However, DILI caused by illicit drug use and guideline-directed management has had little research. We present the case of a 29-year-old female who presented with altered mental status. She was found to have a concomitant liver injury and was treated supportively without the use of NAC, with gradual improvement.

## Introduction

Drug-induced liver injury (DILI) has become an increasingly common condition that affects hepatic metabolism and can progress to liver failure, with accompanying impaired hepatic synthetic function and hepatic encephalopathy [1]. The most common medication to cause acute liver failure is acetaminophen, which accounts for more than 50% of overdose-related cases in the United States [2]. N-acetylcysteine (NAC) is a reversal agent for these cases and is commonly utilized in other drug-induced cases of liver injury; however, it is not a specific treatment for these cases [3]. Although marijuana has not had strong evidence to support it as a cause of acute liver injury, liver injury secondary to cocaine use has become more prevalent; its course is generally self-limiting and commonly resolves rapidly [4-5]. NAC is usually given in cases of liver injury due to its hepatoprotective effects. It is shown to be valuable in non-acetaminophen-induced liver insults such as viral, alcoholic, heat stroke, and mushroom causes due to its ability to reduce glutathione depletion, a vital aspect of proper liver function [6]. There is an essential need to increase clinician awareness of the various forms DILI can present and to establish a guideline-based approach to illicit drug-induced liver injuries with regard to NAC therapy to improve future patient care. Here we present a case of a patient who presented with altered mental status, was found to have severe transaminitis in the setting of polysubstance use, and was treated with supportive management with subsequent improvement of the patient’s clinical status without NAC treatment.

## Case presentation

A 29-year-old female with a past medical history of nonischemic peripartum cardiomyopathy with previously reported ejection fraction (EF) 15-20%, asthma, chronic kidney disease stage 3a, hypertension, and polysubstance use disorder presented to the emergency department (ED) via emergency medical services (EMS) with altered mental status. The patient was initially found unresponsive in a neighboring driveway, with initial medical history and name unable to be obtained at that time due to the patient’s mentation. Per EMS, the patient was found to have a blood glucose of 40 mg/dL and thus received 25 g of dextrose en route with no improvement in mental status. She was found to have a pipe in her belongings, presumably used to smoke crack cocaine. Due to a suspected drug overdose, she received 0.8 mg IV Narcan with a positive response as she was briefly able to regain consciousness before becoming somnolent once again.

Upon arrival at the ED, the patient appeared still lethargic with a Glasgow Coma Score (GCS) of 9. She was subsequently given 1 mg IV Narcan. The patient was then able to respond minimally to sternal rub but still unable to provide medical history. Vital signs on arrival were blood pressure of 91/66 mmHg, heart rate of 122 beats per minute, respiratory rate of 44 breaths per minute, and oxygen saturation of 96%. Initially, the patient was saturating well on room air; however, she subsequently developed increasing oxygen requirements. She was transitioned from nasal cannula to non-rebreather, and then to nasal bilevel airway positive pressure (BiPAP). Pertinent positive physical exam findings were as follows. Generally appeared somnolent with pinpoint pupils bilaterally, and pulmonary exam remarkable for diffuse rhonchi in the bilateral lung fields with increased work of breathing. Initial labs as seen in Table 1 prompted further workup. Venous blood gas revealed pH 7.24, PCO2 44 mmHg, and PO2 88 mmHg on BiPAP.

**Table 1:** Patient's lab values on presentation

	Values	Reference range
Sodium	141 mmol/L	136-145 mmol/L
Potassium	3.6 mmol/L	3.3-4.6 mmol/L
Bicarbonate	15 mmol/L	21-29 mmol/L
Creatinine	2.57 mg/dL	0.51-0.95 mg/dL
Aspartate aminotransferase (AST)	3,428 IU/L	14-33 IU/L
Alanine aminotransferase (ALT)	797 IU/L	10-42 IU/L

**Figure 1:**
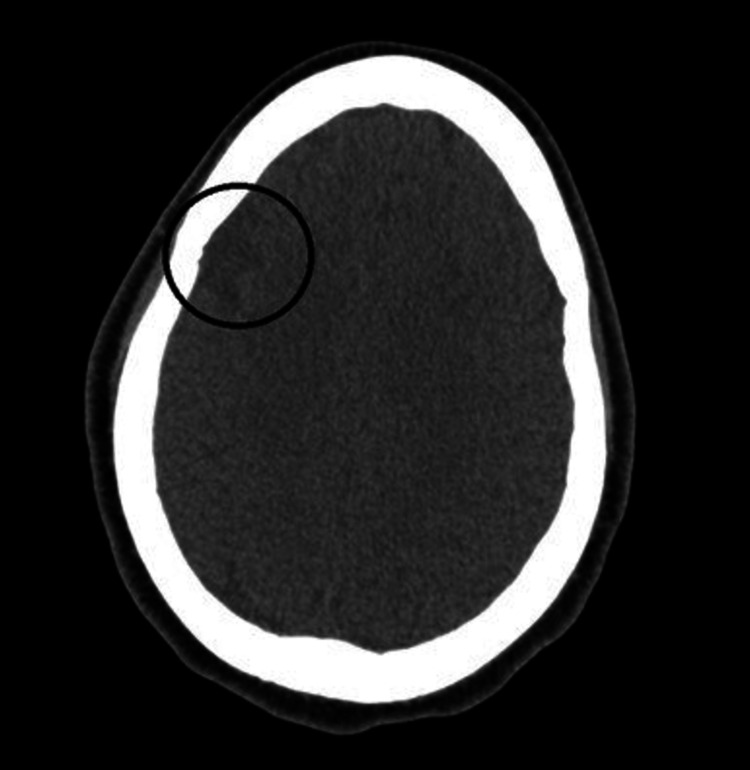
Computed tomography head showing lateral right frontal lobe encephalomalacia (circled)

**Figure 2:**
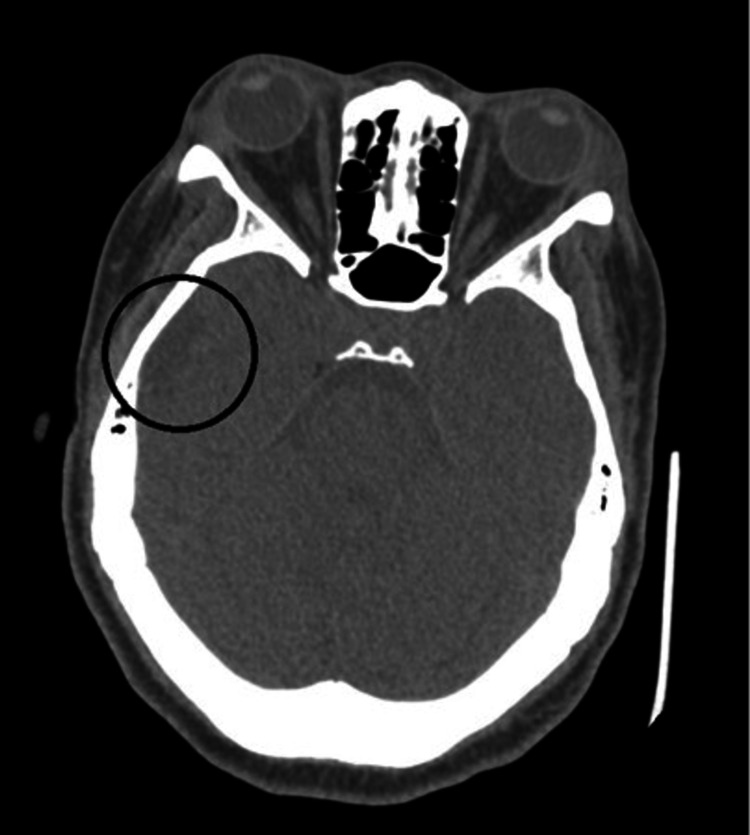
Computed tomography head showing lateral right temporal encephalomalacia

**Figure 3:**
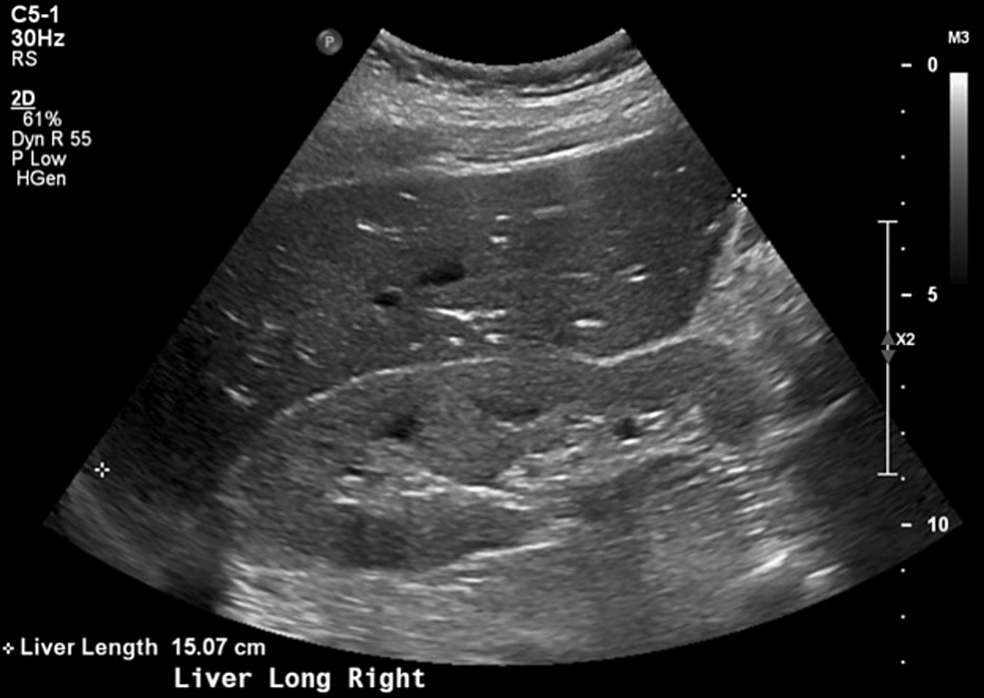
Right upper quadrant ultrasound demonstrating normal echogenicity of the liver with no focal hepatic mass lesions identified

**Figure 4:**
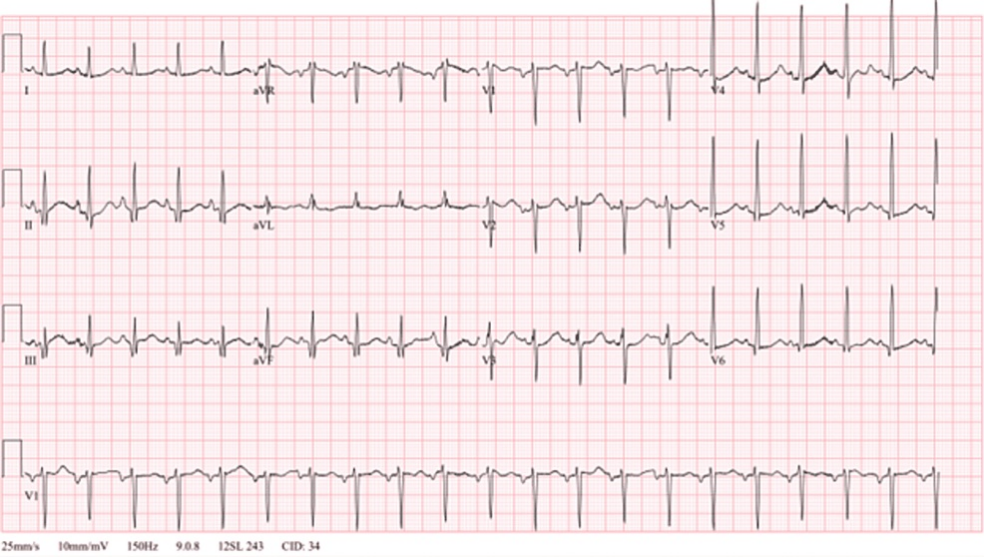
Electrocardiogram showing normal sinus rhythm, heart rate 124 bpm bpm: beat per minute

Urine drug screening was positive for cocaine metabolites, amphetamine, cannabinoids, and fentanyl. Total creatine kinase (CK) was 3,348 U/L indicating rhabdomyolysis. NT-pro BNP was 12,961 pg/mL with a bedside point of care cardiac ultrasound in the ED indicated reduced EF similar to prior. Salicylate and acetaminophen levels were both within normal limits, so NAC was not given. Viral hepatitis panel and human immunodeficiency virus were found to be negative. Ethyl alcohol and alcohol levels were also unremarkable. The trend of the patient’s liver enzymes throughout the admission is shown in Table 2. Computed tomography of the head showed no acute intracranial hemorrhage but was remarkable for encephalomalacia of the lateral right frontal lobe and right temporal lobe (Figures 1, 2). Right upper quadrant ultrasound and chest radiograph showed no acute abnormalities (Figure 3). An electrocardiogram showed sinus tachycardia with a ventricular rate of 124 beats per minute (Figure 4). With this workup, alcoholic hepatitis, viral hepatitis, and acetaminophen-induced liver injury were ruled out. The patient was admitted to the internal medicine floors for toxic encephalopathy secondary to drug overdose with subsequent liver injury and hypercapnic respiratory failure.

**Table 2:** Trend of liver function tests throughout the hospital course AST: aspartate aminotransferase; ALT: alanine aminotransferase

Hospital day	AST (IU/L)	ALT (IU/L)
1	3,428	797
2	5,941	918
3	3,277	705
4	408	370

Initially, the patient was treated with diuretics and albuterol breathing treatments due to suspicion of asthma exacerbations as well as possible systolic heart failure exacerbations causing her dyspnea. The patient’s breathing improved, and she was transitioned to room air while maintaining appropriate oxygen saturation. Due to rhabdomyolysis, acute kidney injury, and drug washout, gentle hydration with intravenous fluids was initiated in the setting of reduced EF with improvement of CK and creatinine. The patient’s mentation throughout the course significantly improved, and her transaminitis trended down. She declined further workups, such as a formal echocardiogram, and was deemed stable for discharge.

## Discussion

There have been few case reports on the effects of cocaine, marijuana, and fentanyl on the liver in clinical practice. Cocaine is a widely used recreational substance that has multi-organ effects. Its general mechanism involves increasing catecholamine production and sympathetic output [7]. Prolonged use of cocaine can thus lead to vasoconstriction causing ischemia in the heart, liver, and kidneys, resulting in a multitude of abnormalities.

Cocaine hepatotoxicity is proposed to occur through two main mechanisms. The first involves cocaine being metabolized to norcocaine in the liver via the cytochrome P450, causing increased stress on this system [8]. The second mechanism is composed of the vasoconstrictive properties of cocaine acting on the venous system of the liver causing hepatic necrosis [8]. On liver histopathology, centrilobular and midzonal necrosis can then be seen [9-10]. Cocaine-induced rhabdomyolysis has been found to negatively affect kidney and liver function, with some researchers suggesting that creatinine can induce the proliferation of reactive oxygen species and activate c-Jun N-terminal kinase, which can contribute to hepatic necrosis and cell injury. This can be indicated by elevations in AST and ALT [10]. While our patient’s drug screen did reveal evidence of fentanyl ingestion, it is believed that it is unlikely that it contributed to this patient’s liver injury. Studies suggest that opioids are very rarely associated with drug-related acute liver injury or hepatic dysfunction when taken in typical pain-relieving dosages [4]. However, the true amount of this patient’s opioid intake is unknown.

Despite its active ingredients being metabolized by the cytochrome P450 system, multiple case reports have failed to show evidence that marijuana is an instigator of acute liver injury [4-5]. Due to the transient rise and subsequent resolution over the next few days of the patient’s hospital course, a diagnosis of cocaine-induced liver injury was made [9]. Most cases of acute liver injury are treated with NAC due to its hepatoprotective effects and preservation of glutathione [6]. However, due to the limited information available with respect to cocaine and NAC treatment, it was not given in this case. Cocaine-induced hepatotoxicity is still a condition with an unclear guideline-directed treatment regimen. Therefore, NAC treatment for non-acetaminophen-induced liver injury is not considered first-line therapy. Thus, further research is warranted on this topic.

## Conclusions

Cocaine-induced hepatotoxicity, an unusual inciting factor for acute liver injury, is becoming increasingly common. It is important for clinicians to be aware of this increasing trend. Treatment approaches involving drug cessation and supportive measures can result in clinical improvement, as exhibited in this case presentation. NAC is generally thought to be hepatoprotective, and further research needs to be done to assess its role in the treatment of non-acetaminophen-induced liver injury.
